# Combined effects of *PNPLA3*, *TM6SF2* and *HSD17B13* variants on severity of biopsy-proven non-alcoholic fatty liver disease

**DOI:** 10.1007/s12072-021-10200-y

**Published:** 2021-06-02

**Authors:** Rafael Paternostro, Katharina Staufer, Stefan Traussnigg, Albert-Friedrich Stättermayer, Emina Halilbasic, Omar Keritam, Elias L. Meyer, Judith Stift, Fritz Wrba, Bence Sipos, Ali Canbay, Martin Schlattjan, Elmar Aigner, Christian Datz, Felix Stickel, Clemens Schafmayer, Jochen Hampe, Stephan Buch, Gerhard Prager, Petra Munda, Mattias Mandorfer, Peter Ferenci, Michael Trauner

**Affiliations:** 1 Division of Gastroenterology and Hepatology, Department of Internal Medicine III, Medical University of Vienna, Waehringer Guertel 18-20, 1090 Vienna, Austria; 2 Department of Visceral Surgery and Medicine, Inselspital, University Hospital Bern, Bern, Switzerland; 3 Center for Medical Statistics, Informatics and Intelligent Systems, Medical University of Vienna, Vienna, Austria; 4 Department of Pathology, Medical University of Vienna, Vienna, Austria; 5 Department of Pathology, Eberhard Karls University Tübingen, Tübingen, Germany; 6 Department of Medicine, Ruhr-Universität Bochum, Bochum, Germany; 7 Department of Gastroenterology and Hepatology, University Hospital Essen, Essen, Germany; 8 First Department of Medicine, Paracelsus Medical University, Salzburg, Austria; 9 Department of Internal Medicine, Oberndorf Hospital, Oberndorf, Austria; 10 Department of Gastroenterology and Hepatology, University Hospital of Zurich, Zürich, Switzerland; 11 Department of General Surgery, University Medicine Rostock, Rostock, Germany; 12 Medical Department 1, University Hospital Dresden, Technische Universität Dresden, Dresden, Germany; 13 Division of General Surgery, Department of Surgery, Medical University of Vienna, Vienna, Austria

**Keywords:** NAFLD, Genetic risk factors, NASH, PNPLA3, TM6SF2, HSD17B13, Fibrosis, Advanced fibrosis, Liver biopsy, Cirrhosis

## Abstract

**Objective:**

Several single-nucleotide polymorphisms have been identified to be disadvantageous or protective in regard to disease severity in patients with non-alcoholic fatty liver disease (NAFLD). However, it is unclear, whether including genetic risk factor(s) either alone or combined into risk stratification algorithms for NAFLD actually provides incremental benefit over clinical risk factors.

**Design:**

Patients with biopsy-proven NAFLD were genotyped for the *PNPLA3*-*rs738409*(minor allele:G), *TM6SF2*-*rs58542926*(minor allele:T) and *HSD17B13*- *rs72613567* (minor allele:TA) variants. The NAFLD activity score (NAS) and fibrosis stage (F0–F4) were used to grade and stage all liver biopsy samples. Patients from seven centers throughout Central Europe were considered for the study.

**Results:**

703 patients were included: NAS ≥ 5:173(24.6%); Fibrosis: F3–4:81(11.5%). *PNPLA3* G/G genotype was associated with a NAS ≥ 5(aOR 2.23, *p* = 0.007) and advanced fibrosis (aOR-3.48, *p* < 0.001).*TM6SF2* T/- was associated with advanced fibrosis (aOR 1.99, *p* = 0.023). *HSD17B13* TA/- was associated with a lower probability of NAS ≥ 5(TA/T: aOR 0.65, *p* = 0.041, TA/TA: aOR 0.40, *p* = 0.033). Regarding the predictive capability for NAS ≥ 5, well-known risk factors (age, sex, BMI, diabetes, and ALT; baseline model) had an AUC of 0.758, Addition of *PNPLA3*(AUC 0.766), *HSB17B13*(AUC 0.766), and their combination(AUC 0.775), but not of *TM6SF2*(AUC 0.762), resulted in a higher diagnostic accuracy of the model. Addition of genetic markers for the prediction of advanced fibrosis (baseline model: age, sex, BMI, diabetes: AUC 0.777) resulted in a higher AUC if *PNPLA3*(AUC 0.789), and TM6SF2(AUC 0.786) but not if *HSD17B13*(0.777) were added.

**Conclusion:**

In biopsy-proven NAFLD, *PNPLA3* G/-, *TM6SF2* T/- and *HSD17B13* TA/- carriage are associated with severity of NAFLD. Incorporating these genetic risk factors into risk stratification models might improve their predictive accuracy for severity of NAFLD and/or advanced fibrosis on liver biopsy.

**Supplementary Information:**

The online version contains supplementary material available at 10.1007/s12072-021-10200-y.

## Introduction

In recent years, several genetic risk factors associated with the susceptibility to and progression of chronic liver disease have been identified [1]. Especially in non-alcoholic fatty liver disease (NAFLD), several genome-wide association studies identified sequence variations in the genes encoding the patatin-like phospholipase domain-containing protein 3 (*PNPLA3*) [2, 3] and transmembrane 6 superfamily member 2 (*TM6SF2*) [4, 5] as risk factors for progressive NAFLD and hepatocellular carcinoma on this background [6]. A variant in the 17B-hydroxysteroid dehydrogenase 13 (*HSD17B13*) gene has been reported to protect against NAFLD, progression from hepatic steatosis to steatohepatitis (NASH), alcoholic liver disease (ALD), and associated liver fibrosis/cirrhosis [7]. Similar observations were reported in patients with hepatitis C virus infection [8], with alcoholic liver disease [9], and with Wilson disease [10]. Furthermore, a study evaluating the combined effect of *PNPLA3* and *TM6SF2* polymorphisms in NAFLD found variants of *PNPLA3* and *TM6SF2* to be independently associated with hepatic steatosis, while *PNPLA3* but not *TM6SF2* was associated with liver fibrosis in a multivariable model. These findings suggest combined disease-modifying effects of these polymorphisms [11].

The aim of our study was to investigate the effects of combining the risk factors *PNPLA3, TM6SF2* and *HSD17B13* on severity of NAFLD/NASH in a large cohort of patients from seven Central European tertiary care medical centers. Furthermore, we investigated if the addition of combined genetic factors to well-known risk factors for NAFLD/NASH increases their predictive value.

## Methods

### Patients

Patients with biopsy-proven non-alcoholic fatty liver disease (NAFLD) from tertiary care medical centers in Austria (Vienna, Oberndorf, Salzburg), Switzerland (Bern) and Germany (Dresden, Kiel, Essen) were included in the study. Standard laboratory markers and detailed information including but not limited to age, height, weight, gender, comorbidities (e.g., diabetes) were recorded at the day of study inclusion. Patients were referred to the respective tertiary care centers with the suspicion of NAFLD (by either steatosis on ultrasound, elevated liver enzymes, presence of metabolic syndrome) and have then undergone confirmatory liver biopsy. Only patients with histologically confirmed NAFLD, graded via the NAFLD activity score (see section “Liver biopsy”), were then included in the study. No patient without liver biopsy was included in the study. Alcohol consumption was examined by self-reporting; subjects with an average alcohol consumption of more than 30 g/day (in men) or 20 g/day (in women) were excluded from further evaluation. Patients with other causes of chronic liver disease (i.e., autoimmune hepatitis, PSC/PBC, Wilson Disease, Hemochromatosis) were all excluded from the study. Patients with active viral hepatitis (HBs-Antigen positivity, detectable HBV-DNA, detectable HCV-RNA) were also excluded from the study—however, Anti-HBc- or Anti-HCV-Antibody-positive patients were included if their biopsy results clearly stated NAFLD as the primary cause of their liver disease.

Further exclusion criteria were: incomplete or missing liver biopsy results, missing blood samples for genotyping, and missing informed consent. Finally, seven hundred and three patients were included in the study.

### Liver biopsy

Percutaneous liver biopsies were obtained in all patients using a Menghini needle (Hepafix, B. Braun Melsungen, Melsungen, Germany) within clinical routine for diagnostic reasons. Biopsy samples were routinely processed (formalin fixed and paraffin embedded) and stained with hematoxylin/eosin and chromatrope aniline blue for assessment of hepatic steatosis, liver fibrosis, and hepatic inflammation.

Hepatic steatosis was graded according to Brunt et al. [14] using a three-point scale by calculating the percentage of lipid containing hepatocytes at a 40 × magnification: mild (G1: 5–33%), moderate (G2: 34–66%), or severe steatosis (G3: > 66%). According to the study design, patients with less than 5% of fat containing hepatocytes (G0) were excluded from our analysis. Hepatocyte ballooning was graded as absent (0), rare (1), or prominent ballooning (2). Necro-inflammatory activity was graded as absent (0), mild (1), moderate (2), or severe (3). The NAFLD activity score (NAS) [12, 13] was calculated as the sum of steatosis (1–3), hepatocyte ballooning (0–2), and inflammation (0–3) as a score from 1 to 8. Liver fibrosis was staged on a five-point scale: no fibrosis (stage 0), pericellular fibrosis (stage 1), pericellular and portal fibrosis (stage 2), bridging fibrosis (stage 3), or cirrhosis (stage 4) [12]. Since severity of NAFLD is mostly characterized by the presence/absence of steatohepatitis, we defined “severe NAFLD” as patients having a NAS ≥ 5 according to studies showing excellent discriminative value for this cutoff for the presence of definite NASH [12, 13].

### Genotyping

*HSD17B13-rs72613567*, *PNPLA3-rs738409* and *TM6SF2-rs58542926* genotyping was performed by StepOnePlus Real-Time PCR System and a TaqMan SNP Genotyping Assay (Applied Biosystems, Foster City, CA, USA).

### Statistical analysis

Please refer to the supplemental material for a detailed description of the statistics used in this manuscript.

### Ethics

The study was approved by the ethics committee of the Medical University of Vienna (EK 747/2011) and the local ethics committees of the participating centers and performed in accordance with the current version of the Helsinki Declaration. All patients signed an informed consent form prior to study inclusion.

## Results

Within the 703 included patients with biopsy-proven NAFLD, distribution of NAS was as follows: NAS 1–2—328 (46.7%), NAS 3–4—202 (28.7%) and NAS ≥ 5—173 (24.6%). Advanced fibrosis (fibrosis stage 3 or 4) was present in 81 (11.5%) and cirrhosis (F4) in 29 (4.1%) of patients. For further patient characteristics, see Supplementary Table 1.

### PNPLA3 and severity of non-alcoholic fatty liver disease

Carriage of at least one G-allele was associated with a more severe phenotype in regard to NAS and presence of advanced fibrosis (Supplementary Table S1). G-allele carriers were more often found to have a NAS ≥ 5 (G/G: 33 [42.3%] vs. C/G 75 [28.1%] vs. C/C 65 [18.2%], *p* < 0.001) and severe steatosis (grade 3: G/−: 118 [34.2%] vs. C/C: 64 [17.9%], *p* < 0.001). Grade 2 ballooning was significantly more often seen in G-allele carriers (G/−: 60 [17.4%] vs. C/C: 37 [10.3%], *p* = 0.007), while severity of inflammation was not different between G-allele carriers and C/C genotypes (*p* = 0.676). Advanced fibrosis was significantly more common in G-allele carriers (G/−: 50 [14.5%] vs. C/C: 31 [8.7%], *p* = 0.015) with G/G-carriers having the highest prevalence (G/G: 24.4% vs. C/G: 11.6% vs. C/C: 8.7%, Supplemental Table S1). Both median AST (*p* < 0.001) and ALT (*p* < 0.001) were significantly higher in G-allele carriers (Supplementary Table S1).

Multivariable binary logistic regression analysis found PNPLA3 G/G carriage associated with NAS ≥ 5 (aOR: 2.23, *p* = 0.007) and F ≥ 3 (aOR: 3.48, *p* < 0.001) independent of presence of diabetes, ALT and gender (for NAS ≥ 5) and of age and presence of diabetes (for F ≥ 3; for aORs see Tables 1A and 2A).

**Table 1:** Uni- and multivariable binary regression analyses of clinical and laboratory markers associated with *NAS* ≥ *5* on liver biopsy and adjusted for (A) PNPLA3 genotype, (B) TM6SF2 genotype, (C) HSD17B13 genotype and (D) “risk-allele” model (one point for each risk allele, e.g., PNPLA3 G-Allele, TM6SF2 T-Allele, HSD17B13 T-Allele; groups “no risk alleles” and “1 risk allele” have been subsumed into one group; to overcome potential effects of dilution by combining risk polymorphisms [PNPLA3,TM6SF2] with protective polymorphisms [HSD17B13], HSD17B13 T-Allele carriage was classified as the “risk allele” [rather than TA-Allele carriage as “protective”] for the combined “risk-allele” model)

	Univariable			Multivariable		
	OR	95%CI	*p*-value	aOR	95%CI	*p*-value
A						
Age, per year	1.00	0.99–1.02	0.529	1.00	0.99–1.02	0.660
Sex, for being male	1.03	0.73–1.46	0.848	0.57	0.38–0.86	0.007
BMI, per unit	0.98	0.96–0.99	0.002	0.99	0.98–1.01	0.490
Diabetes, yes vs. no	1.66	1.16–2.37	0.005	1.76	1.17–2.67	0.007
ALT, per unit	1.02	1.02–1.03	< 0.001	1.02	1.02–1.03	< 0.001
PNPLA3 genotype						
C/C vs. C/G	1.76	1.21–2.57	0.003	1.45	0.96–2.19	0.075
G/G	3.31	1.96–5.58	< 0.001	2.23	1.25–3.99	0.007
B						
Age, per year	–	–	–	1.00	0.98–1.02	0.690
Sex, for being male	–	–	–	0.55	0.37–0.83	0.004
BMI, per unit	–	–	–	0.99	0.97–1.01	0.402
Diabetes, yes vs. no	–	–	–	1.79	1.19–2.70	0.005
ALT, per unit	–	–	–	1.02	1.02–1.03	< 0.001
TM6SF2 genotype						
C/C vs. T/–	1.58	1.02–2.44	0.041	1.51	0.94–2.44	0.091
C						
Age, per year	–	–	–	1.00	0.99–1.02	0.860
Sex, for being male	–	–	–	0.57	0.38–0.86	0.007
BMI, per unit	–	–	–	0.99	0.97–1.01	0.406
Diabetes, yes vs. no	–	–	–	1.80	1.19–2.72	0.005
ALT, per unit	–	–	–	1.02	1.02–1.03	< 0.001
HSD17B13 genotype						
T/T vs. T/TA	0.66	0.46–0.96	0.031	0.65	0.43–0.98	0.041
TA/TA	0.43	0.20–0.94	0.034	0.40	0.17–0.93	0.033
D						
Age, per year	–	–	–	1.00	0.98–1.02	0.702
Sex, for being male	–	–	–	0.57	0.38–0.87	0.009
BMI, per unit	–	–	–	0.99	0.97–1.02	0.666
Diabetes, yes vs. no	–	–	–	1.79	1.18–2.72	0.007
ALT, per unit	–	–	–	1.02	1.02–1.03	< 0.001
Risk Alleles (Number of)						
0–1 Alleles vs. 2	2.19	1.24–3.86	0.007	2.22	1.19–4.11	0.012
3	3.53	1.98–6.31	< 0.001	3.42	1.81–6.46	< 0.001
4	5.65	2.83–11.23	< 0.001	4.65	2.17–9.95	< 0.001
5	5.11	1.63–16.04	0.005	3.24	0.85–12.29	0.084

**Table 2:** Uni- and multivariable binary regression analyses of clinical and laboratory markers associated with *F* ≥ *3* on liver biopsy and adjusted for (A) PNPLA3 genotype, (B) TM6SF2 genotype, (C) HSD17B13 genotype and (D) “risk-allele” model (one point for each risk allele, e.g., PNPLA3 G-Allele, TM6SF2 T-Allele, HSD17B13 T-Allele; groups “no risk alleles” and “1 risk allele” have been subsumed into one group; to overcome potential effects of dilution by combining risk polymorphisms [PNPLA3,TM6SF2] with protective polymorphisms [HSD17B13], HSD17B13 T-Allele carriage was classified as the “risk allele” [rather than TA-Allele carriage as “protective”] for the combined “risk-allele” model)

	Univariable			Multivariable		
	OR	95%CI	*p*-value	aOR	95%CI	*p*-value
A						
Age, per year	1.06	1.04–1.08	< 0.001	1.04	1.02–1.07	< 0.001
Sex, for being male	1.69	1.06–2.69	0.028	1.48	0.88–2.47	0.134
BMI, per unit	0.95	0.93–0.97	< 0.001	0.98	0.95–1.001	0.057
Diabetes, yes vs. no	3.91	2.42–6.31	< 0.001	3.36	1.98–5.69	< 0.001
PNPLA3 genotype						
C/C vs. C/G	1.39	0.82–2.34	0.224	1.51	0.85–2.65	0.155
G/G	3.40	1.80–6.41	< 0.001	3.48	1.73–6.96	< 0.001
B						
Age, per year	–	–	–	1.04	1.02–1.06	< 0.001
Sex, for being male	–	–	–	1.43	0.86–2.38	0.167
BMI, per unit	–	–	–	0.97	0.95–0.99	0.025
Diabetes, yes vs. no	–	–	–	3.45	2.05–5.82	< 0.001
TM6SF2 genotype						
C/C vs. T/–	1.82	1.05–3.15	0.033	1.99	1.10–3.62	0.023
C						
Age, per year	–	–	–	1.04	1.02–1.06	0.001
Sex, for being male	–	–	–	1.44	0.86–2.38	0.160
BMI, per unit	–	–	–	0.97	0.94–0.99	0.016
Diabetes, yes vs. no	–	–	–	3.35	2.00–5.64	< 0.001
HSD17B13 genotype						
T/T vs. T/TA	0.81	0.49–1.33	0.406	0.86	0.50–1.48	0.596
TA/TA	0.70	0.27–1.83	0.462	0.84	0.30–2.31	0.734
D						
Age, per year	–	–	–	1.04	1.02–1.06	< 0.001
Sex, for being male	–	–	–	1.46	0.87–2.45	0.150
BMI, per unit	–	–	–	0.98	0.95–1.001	0.060
Diabetes, yes vs. no	–	–	–	3.44	2.02–5.85	< 0.001
Risk Alleles (Number of)						
0–1 Alleles vs. 2	2.31	1.04–5.17	0.041	2.21	0.96–5.12	0.064
3	2.59	1.13–5.98	0.025	2.61	1.09–6.26	0.032
4	4.54	1.78–11.56	0.002	4.34	1.61–11.66	0.004
5	12.33	3.52–43.23	< 0.001	11.57	2.91–46.07	0.001

### TM6SF2 and severity of non-alcoholic fatty liver disease

Absolute numbers of T-allele carriers were quite low (*n* = 115 [16%]). The severity of steatosis (percent hepatocytes with lipid accumulation) was significantly higher in T-allele carriers (*p* < 0.001, Supplementary Table S2). Neither ballooning nor inflammation grade differed between C/C and T/− allele carriers. However, advanced fibrosis was significantly more often found in T/− carriers (T/−: 20 [17.4%] vs. C/C: 61 [10.4%], *p* = 0.031). Finally, both median AST (*p* = 0.040) and ALT (*p* = 0.034) levels were higher in T/− carriers (Supplementary Table S2).

Multivariable binary logistic regression analysis found TM6SF2 T/− allele carriage associated with advanced fibrosis (aOR: 1.99, *p* = 0.023) independent of age, BMI and diabetes mellitus (see Table 2B for aORs). While T-Allele carriage was associated with NAS ≥ 5 in univariable analysis (OR 1.58, *p* = 0.041), this effect was not seen after adjusting for other variables in the multivariable model (aOR: 1.51, *p* = 0.091; Tables 1B and 2B).

### HSD17B13 and severity of non-alcoholic fatty liver disease

Significantly less TA-allele carriers were found with NAS ≥ 5 (TA/TA 8 [14.5% [vs. TA/T 52 [20.8%] vs. T/T 113 [28.4%], *p* = 0.018; Supplementary Table S3). No statistical difference was seen in respect to degree of steatosis (*p* = 0.424), inflammation (*p* = 0.127) or ballooning (*p* = 0.123) for patients carrying the protective *HSD17B13* TA/− variant. TA-allele carriage was not associated with a milder phenotype in regard to presence of advanced fibrosis (TA/−: 31 [10.2%] vs. T/T: 50 [12.6%], *p* = 0.324) or cirrhosis (TA/−: 10 [3.3%] vs. T/T: 19 [4.8%], *p* = 0.323; Supplementary Table S3).

Multivariable binary logistic regression analysis revealed both T/TA and TA/TA genotypes associated with a lower probability of NAS ≥ 5 (T/TA aOR: 0.65, *p* = 0.041, TA/TA aOR: 0.40, *p* = 0.033) independent of gender, diabetes and ALT (for aORs see Table 1C). TA-Allele carriage was, however, not associated with a lower probability for advanced fibrosis (Table 2C).

### Combination effects of PNPLA3, TM6SF2 and HSD17B13 on disease severity

To test single- and combined effects of each genetic polymorphism on disease severity at clinical presentation, we performed a stepwise approach to elucidate whether the addition of single/combined genetic markers improve diagnostic performance. Clinical endpoints were either presence of NAS ≥ 5 (model 1), advanced fibrosis (F3/4) (model 2) or significant fibrosis (*F* ≥ 2) (model 3). The evidence of NASH on liver biopsy (≥ NAS 5) “baseline model” included age, sex, BMI, diabetes, and ALT. The advanced/significant fibrosis “baseline model” included age, sex, BMI, and diabetes.

**Fig. 1:**
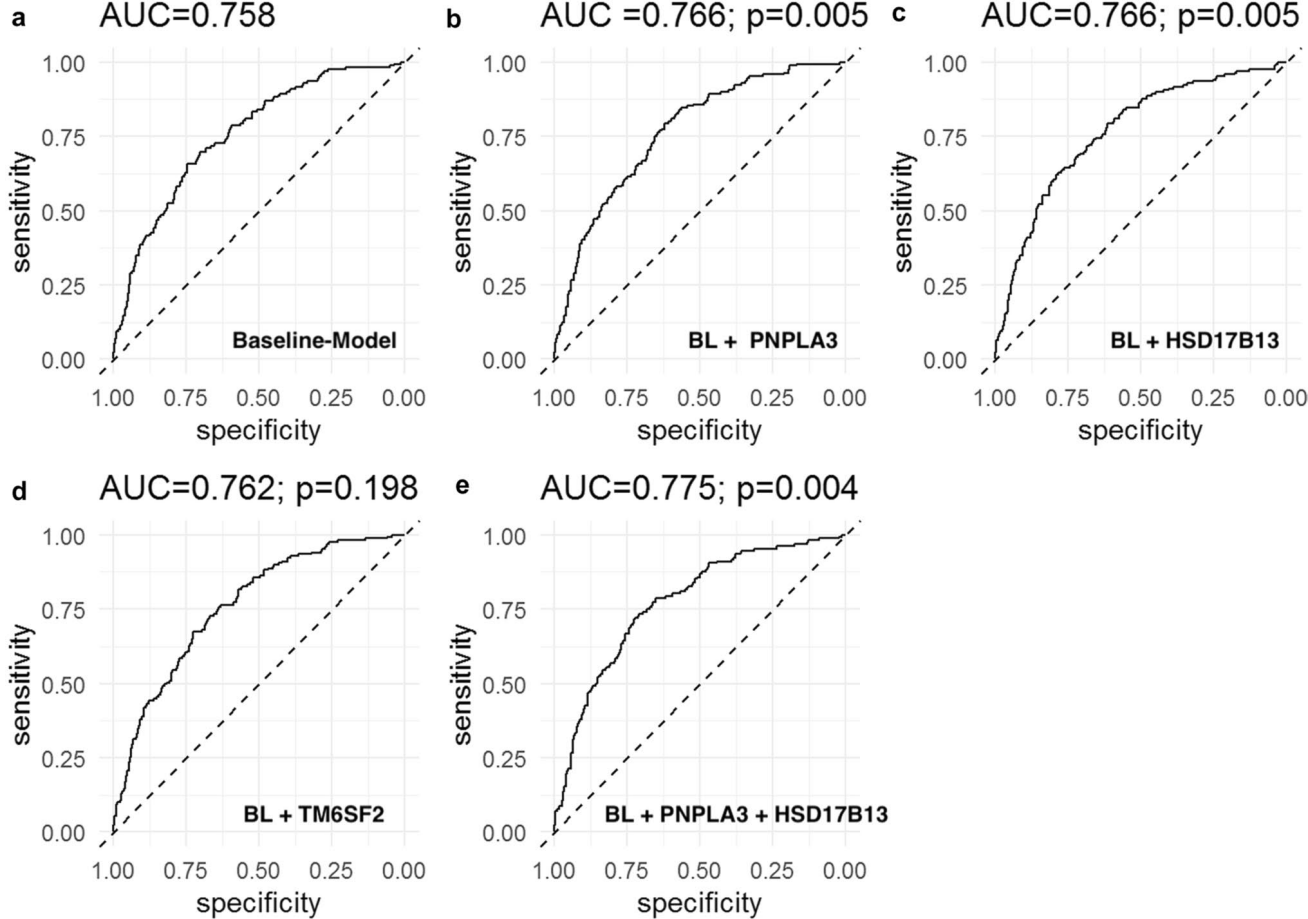
AUC for predicting NAS ≥ 5 applying **a** baseline model (BL; age, sex, BMI, diabetes, and ALT), **b** BL + PNPLA3, **c** BL + HSD17B13, **d** BL + TM6SF2, and **e** BL + PNPLA3 + HSD17B13; *P*-values: **b**, **c**, **d** vs. BL model; **e** vs. PNPLA3 model

In regard to prediction of NAS ≥ 5 on liver biopsy, the AUC of the baseline model was 0.758 (Fig. 1). Addition of *PNPLA3* (AUC 0.766, *p* = 0.005 vs. BL) and *HSD17B13* (AUC 0.766, *p* = 0.005 vs. BL) genotypes resulted in improved AUC. In contrast, addition of *TM6SF2* to BL did not improve AUC (AUC 0.762, *p* = 0.198). Finally, addition of both *PNPLA3* and *HSD17B13* resulted in the highest diagnostic accuracy (AUC 0.775) and this was significantly better compared to the *PNPLA3* model alone (*p* = 0.004; Fig. 1).

**Fig. 2:**
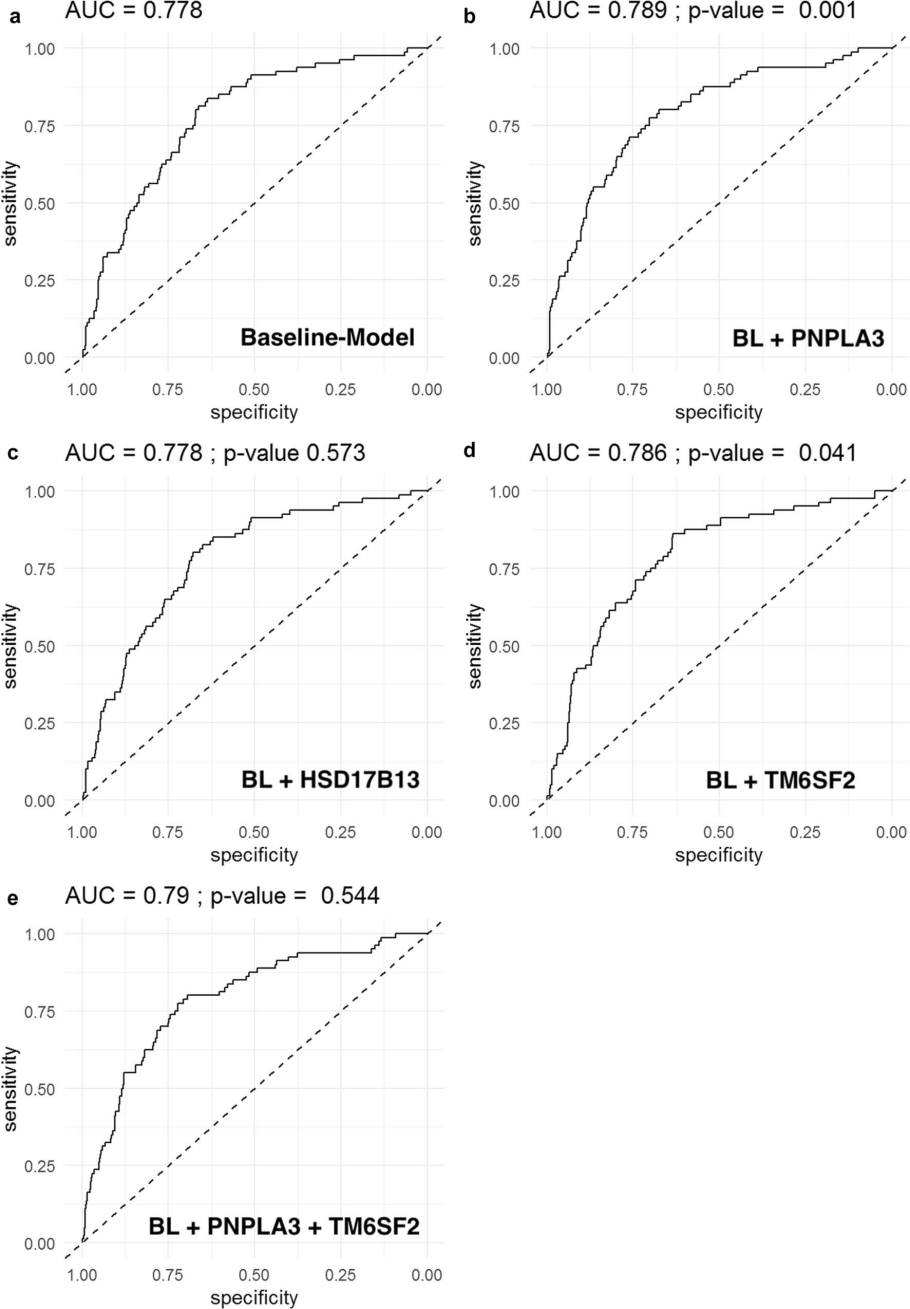
AUC for predicting advanced fibrosis (≥ F3) using **a** baseline model (BL; age, sex, BMI, and diabetes), **b** BL + PNPLA3, **c** BL + HSD17B13 **d**, BL + TM6SF2, **e** BL + PNPLA3 + TM6SF2; *P*-values: **b**, **c**, and **d** vs. BL model; **e** vs. PNPLA model)

The AUC using the baseline (BL) model for advanced fibrosis was 0.778 (Fig. 2). Addition of *PNPLA3* (AUC 0.789, *p* = 0.001 vs. BL), *TM6SF2* (0.786, *p* = 0.041 vs. BL) but not *HSD17B13* (AUC 0.777, *p* = 0.696 vs. BL) resulted in a higher diagnostic AUC. Finally, addition of both *PNPLA3-* and *TM6SF2* genotypes to the BL model did not significantly improve the AUC (AUC 0.79, *p* = 0.544), when compared to the best model including only one genetic risk factor (PNPLA3 model).

Prediction of significant fibrosis (*F* ≥ 2) showed an AUC of 0.76 for the baseline model (Supplemental Figure S4). Only addition of PNPLA3 status significantly improved the model (AUC 0.774, *p* = 0.001).

**Fig. 3:**
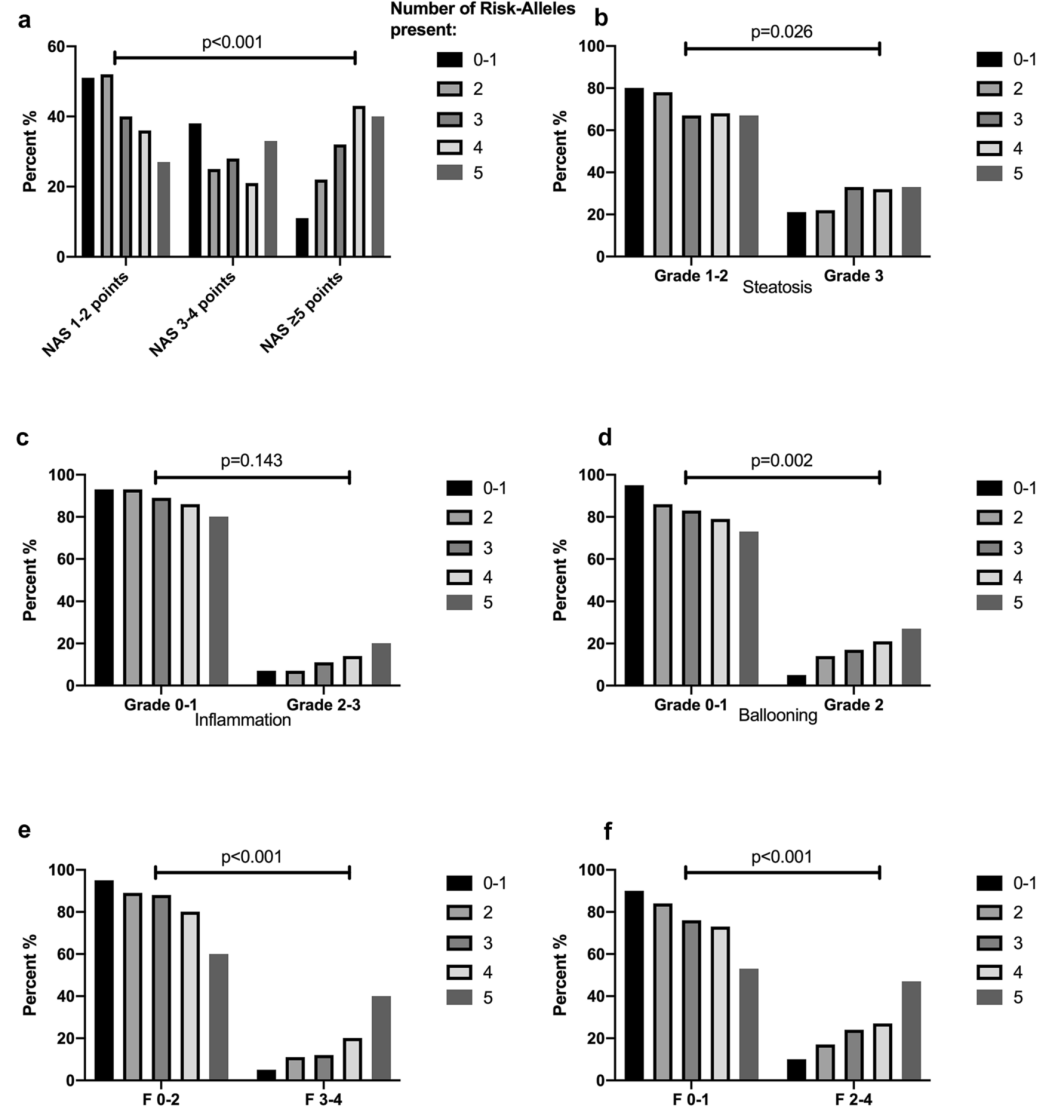
Distribution of risk alleles (PNPLA3 G, TM6SF2 T and HSD17B13 T-allele) across disease severity strata (**a**—NAS; **b**—Steatosis; **c**—Inflammation; **d**—Ballooning; **e**—*F* ≥ 3; **f**—*F* ≤ 2)

Finally, to further investigate the risk-allele approach we analyzed clinical markers of disease severity in patients stratified according to the number of risk alleles carried. Here, we found that prevalence of NAS ≥ 5, severe steatosis, ballooning and presence of advanced or significant fibrosis and even cirrhosis significantly increased in an “allele dose”-dependent manner (Tables 1D, 2D, 3 and Fig. 3). Furthermore, multivariable binary logistic regression analysis found the “risk-allele” model associated with NAS ≥ 5 and advanced fibrosis (*F* ≥ 3) independent of gender, diabetes and ALT (for NAS ≥ 5) and age and diabetes (for *F* ≥ 3) also in an “allele dose”-dependent manner (refer to Tables 1D and 2D for adjusted ORs).

**Table 3:** Patient characteristics stratified by the number of PNPLA3, TM6SF2 and HSD17B13, risk alleles “(minimum: 0–1, maximum: 5; no patient was found with all 6 “risk alleles”)

	0–1 Alleles	2 Alleles	3 Alleles	4 Alleles	5 Alleles	*p*-value
NAS, *n* (%)						< 0.001
1–2 Points	79 (50.6%)	146 (52.3%)	75 (40.1%)	24 (36.4%)	4 (26.7%)	
3–4 Points	59 (37.8%)	71 (25.4%)	53 (28.3%)	14 (21.2%)	5 (33.3%)	
≥ 5 points	18 (11.5%)	62 (22.2%)	59 (31.6%)	28 (42.4%)	6 (40%)	
NAS, *n* (%)						< 0.001
1–4 Points	138 (88.5%)	217 (77.8%)	128 (68.4%)	38 (57.6%)	9 (60%)	
≥ 5 points	18 (11.5%)	62 (22.2%)	59 (31.6%)	28 (42.4%)	6 (40%)	
NAS Steatosis, *n* (%)						0.026
Grades 1–2	124 (79.5%)	217 (77.8%)	125 (66.8%)	45 (68.2%)	10 (66.7%)	
Grade 3	32 (20.5%)	62 (22.2%)	62 (33.2%)	21 (31.8%)	5 (33.3%)	
NAS Inflammation, *n* (%)						0.143
Grades 0–1	145 (92.9%)	259 (92.8%)	166 (88.8%)	57 (86.4%)	12 (80%)	
Grades 2–3	11 (7.1%)	20 (7.2%)	21 (11.2%)	9 (13.6%)	3 (20%)	
NAS Ballooning, *n* (%)						0.002
Grades 0–1	148 (94.9%)	240 (86%)	155 (82.9%)	52 (78.8%)	11 (73.3%)	
Grade 2	8 (5.1%)	39 (14%)	32 (17.1%)	14 (21.2%)	4 (26.7%)	
Fibrosis, *n* (%)						< 0.001
Grades 0–1	141 (90.4%)	233 (83.5%)	142 (75.9%)	48 (72.7%)	8 (53.3%)	
Grades 2–4	15 (9.6%)	46 (16.5%)	45 (24.1%)	18 (27.3%)	7 (46.7%)	
Fibrosis, *n* (%)						< 0.001
Grades 0–2	148 (94.9%)	248 (88.9%)	164 (87.7%)	53 (80.3%)	9 (60%)	
Grades 3–4	8 (5.1%)	31 (11.1%)	23 (12.3%)	13 (19.7%)	6 (40%)	
Cirrhosis, *n* (%)						< 0.001
No	153 (98.1%)	268 (96.1%)	179 (95.7%)	63 (95.5%)	11 (73.3%)	
Yes	3 (1.9%)	11 (3.9%)	8 (4.3%)	3 (4.5%)	4 (26.7%)	

To overcome potential effects of dilution by combining risk polymorphisms [PNPLA3,TM6SF2] with protective polymorphisms [HSD17B13], HSD17B13 T-Allele carriage was classified as the “risk allele” [rather than TA-Allele carriage as “protective”] for the combined “risk-allele” model)

## Discussion

In this study, we analyzed the impact of combined genetic variants in *PNPLA3*, *TM6SF2* and *HSD17B13* on severity and phenotype of NAFLD/NASH in a large multi-center cohort of patients with biopsy-proven NAFLD.

First, we confirmed that harboring at least one *PNPLA3* G-allele was associated with a more advanced phenotype in regard to both presence of NAS ≥ 5 and advanced fibrosis on liver biopsy*. TM6SF2* T-allele carriage, even though the absolute number of T-allele carriers was low, was also associated with a more severe phenotype in regard to advanced fibrosis. Conversely, *HSD17B13* TA/- allele carriage was associated with a milder NAFLD phenotype.

Second, we could show that combining all three genetic variants to a “risk-allele” model was associated with a higher probability for NAS ≥ 5 and advanced fibrosis in an allele-dose-dependent manner—meaning the more risk alleles, the higher the risk for severe NAFLD/NASH at diagnosis.

Thirdly, we found that addition of *PNPLA3* and *HSD17B13* genotypes, alone and in combination, to well-known clinical risk factors for NASH resulted in better accuracy to non-invasively predict a NAS ≥ 5. Interestingly, an “allele-dose”-dependent effect was observed. In regard to presence of advanced fibrosis (≥ F3), both *PNPLA3* and *TM6SF2* models resulted in higher AUC than the baseline model; however, when significant fibrosis (≥ F2) was the endpoint of interest, only *PNPLA3 *status remained significant. Nevertheless, while significant, those “addition” effects were only modest, highlighting the importance of standard clinical indices (age, diabetes, ALT, etc.) in the diagnostic work-up of NAFLD.

The effects of the *PNPLA3-I148M* variant on NAFLD/NASH severity have been extensively studied [2, 15]. In previous studies, the rs738409 C > G variant increased the risk for hepatic steatosis, liver fibrosis/cirrhosis, as well as hepatocellular carcinoma in a wide spectrum of liver disease [16]. The *PNPLA3* protein is highly expressed in the liver and has lipase activity towards triglycerides in hepatocytes and towards retinyl esters in hepatic stellate cells (HSC) [17]. The I148M mutation leads to loss of function resulting in hepatic fat and retinol retention [2]. *PNPLA3* is required for human HSC activation and the *I148M* variant confers proinflammatory and profibrogenic properties to HSCs, leading to increased proliferation and migration, as well as production and release of cytokines and chemokines, which amplifies liver injury [17–19].

The pathophysiological role of the *HSD17B13* variant and its protective effect on chronic liver disease is not yet fully understood and connecting the genetic association with a pathophysiological explanation requires elucidation. Moreover, the impact of *HSD17B13* on the clinical course of liver disease could be stage dependent [20]. In general, 17BHSD are enzymes catalyzing the conversion between 17-keto and 17-hydroxysteroids [21]. Fifteen HSDs have been identified and most of them play a role in regulation of biological activity of sex hormones [21], while *HSD17B13* has been found to be merely involved in sex hormone metabolism [21, 22]. *HSD17B13* is a hepatic retinol-dehydrogenase (RDH) involved in retinoid homeostasis [23]. Interestingly, hepatic expression of *HSD17B13* was 5.9 times higher in NASH patients, although *HSD17B13* genotype (rs6834314 or rs72613567) did not affect hepatic expression [23].

Finally, *TM6SF2* T/- allele carriage has been linked to disease severity in NAFLD [4], but interestingly, also to a lower risk for cardiovascular endpoints [24]. It is thought that the loss-of-function *TM6SF2* T/- polymorphism leads to a higher liver triglyceride content in hepatocytes, while lowering circulating lipoproteins [25].

Whether the addition of genetic markers to well-known, clinically practicable, risk factors for NASH and advanced fibrosis facilitates risk stratification is yet unknown. In our study, the addition of both *PNPLA3* and *HSD17B13* genotypes significantly improved the accuracy for the non-invasive prediction of a NAS ≥ 5; however, the *PNPLA3 model* was most robust in the prediction of advanced and significant fibrosis when added to well-known clinical factors. Despite *TM6SF2* showing improved AUCs compared to the baseline model, addition of *TM6SF2* to the *PNPLA3* “only” model did not significantly improve AUC and might, therefore, be uncalled-for. Moreover, we were able to demonstrate that throughout almost all histological markers of NAFLD (NAS including subgroups, fibrosis stage), disease severity increased in an “allele-dose”-dependent manner, as the severity of liver disease showed a stepwise increase with increasing number of risk alleles. Finally, wherever genetic testing is available, the potential applicability of our results in clinical practice should be high since patients from several (total of 7) centers were included in the study; hence, our cohort very well represents the average Central European NAFLD patient.

Nevertheless, when added on top of clinical risk factors, the improvements in AUC were moderate, suggesting that the clinical impact of genetic testing for assessment of disease severity may be limited. We would like to point out that in addition to other well-established risk factors, we also included ALT in the models for predicting NASH, which by itself is determined by the genetic background (as indicated by our and previous studies [26]) and thus, may reduce the impact of genetic factors if considered in the same model. However, since ALT is readily available in NAFLD patients, we decided to add it to the BL risk stratification model. Most importantly, we additionally evaluated whether addition of the respective genetic markers improved diagnostic accuracy for well-known non-invasive fibrosis scores such as FIB-4 or the NAFLD Fibrosis score (NFS) and could show that addition of PNPLA3 improved accuracy of only the NFS but not FIB-4 (Supplemental Figure S5 and S6). HSD17B13 and TM6SF2 both did not show significant improvements of the respective AUCs. However, data regarding non-invasive fibrosis scores were not available in all patients and therefore, this finding still needs further evaluation.

Previous studies have looked into the concept of combining genetic risk factors and thereby improving their accuracy in predicting disease severity [27, 28]. Most recently, a combination of our proposed three genetic risk markers (PNPLA3, TM6SF2, HSD17B13) into a genetic risk score was shown to be associated with an increased risk for cirrhosis and HCC in the general population [29]—further emphasizing the clinical feasibility to utilize those combined markers also, and especially, in diagnosed NAFLD patients. Most recently, Anstee et al. found PNPLA3, TM6SF2, GCKR and HSD17B13 to have genome-wide significance on disease severity in NAFLD in an elegantly conducted GWAS study [30]. However, they did not assess the impact on clinical feasibility in non-invasively staging/predicting severity of NAFLD and did not implement a genetic risk score.

The major limitations of our study are the lack of non-invasive biomarkers and of longitudinal data. Also, transient elastography or other non-invasive imaging parameters for NAFLD (e.g., magnetic resonance imaging/elastography[MRI/MRE]) were not available in our patients. Especially in regard to imaging data, the question whether combining genetic information with MRE data could improve its diagnostic accuracy might be worth studying and should be focus of future studies, especially since several studies have shown excellent AUCs for MRE to non-invasively predict fibrosis, not only in adults but also in children [31–33]. Moreover, information on *MBOAT7* genotype and other genetic factors was not available in most patients. MBOAT7 was, however, not associated with a more severe phenotype in the (small) subgroup of patients where data was available (data not shown). These limitations may be outweighed by strengths of our study using liver biopsy (still the gold standard for fibrosis assessment) in a cohort of NAFLD patients from across Central Europe with information on genetic data on three important risk factors of NAFLD (*PNPLA3*, *HSD17B13*, and *TM6SF2*). An ideal study should explore the role of genetic risk factors in patients having in addition to liver biopsy a whole array of non-invasive markers. In clinical practice, outside of clinical trials, the population for non-invasive tests would be patients with only ultrasound and clinical data. Thus, the accuracy of our model should also be tested in this specific population.

In conclusion, in biopsy-proven NAFLD, *PNPLA3-I148M G/−, TM6SF2 T/−* and *HSD17B13 TA/−* carriage are associated with severity of NAFLD in an “allele-dose”-dependent fashion. The addition of genetic factors and their combinations into risk stratification models might provide incremental information and improve their predictive accuracy for severity of NAFLD (NAS ≥ 5) and/or advanced/significant fibrosis on liver biopsy.

## Supplementary Information

Below is the link to the electronic supplementary material.

Supplementary file1 (DOCX 438 kb)
