# Supplementary material for: Combined effects of PNPLA3, TM6SF2 and HSD17B13 variants on severity of biopsy-proven non-alcoholic fatty liver disease
Source: Hepatol Int. 2021 Jun 2;15(4):922–33. doi: 10.1007/s12072-021-10200-y (PMC8382644; doi:10.1007/s12072-021-10200-y)

*Statistical Analysis*

Continuous variables were reported as mean ± standard deviation (SD) or median (IQR), and categorical variables were reported as number (n) of patients with the certain characteristic (proportion of patients with the certain characteristics [%]). Student’s t-test was used for group comparisons of normally distributed data, while Mann-Whitney-U test was applied when data was not normally distributed. Pearson's chi-squared test or Fisher’s exact test were performed to conduct group comparisons of categorical variables. The prognostic value of the genetic risk factors additionally to routinely assessed patient variables that are clinically relevant and have previously been associated with increased risk (further referred to as “baseline variables”) was assessed using logistic regression models. For each of the three outcomes: advanced fibrosis (F3/4), significant fibrosis (≥F2) and NAS ≥5, we fitted a “baseline model” including only “baseline variables” (Variables: advanced fibrosis F3/4; significant fibrosis ≥F2: age, sex, BMI, diabetes; NAS ≥5: age, sex, BMI, diabetes, ALT) and subsequently included each of the genetic risk factors separately. Improved model fit was assessed using a likelihood-ratio test and genetic risk factors significantly improving the model fit compared to the “baseline model” were included in a final model. Model fit was visualized using receiver operating characteristics curves and the corresponding area-under the receiver operating characteristics curve, alongside p-values from the likelihood ratio test, which refer to improvement in model fit. Furthermore uni- and multivariable binary logistic regression models were conducted to evaluate factors independently associated with NAS≥5 or F≥3 respectively. Two-sided p values <0.05 were considered as statistically significant. Since p-values serve only descriptive purposes, no multiplicity correction was applied. The IBM SPSS 24.0 statistic software (SPSS Inc., Armonk, NY) and R 3.6.3[Package: ggplot2, pROC](19) were used for all statistical analyses. All analyzes were performed by Rafael Paternostro (clinical researcher and medical doctor, ≥5 year experience in the field of clinical research) and Elias L. Meyer (Professional Statistican, Medical University of Vienna).

**Suppl. Table S1:** Patient characteristics of the overall study population and patient characteristics stratified according to PNPLA3 status.

|  | **All patients (n=703)** | **C/C (n=358)** | **C/G (n=267)** | **G/G (n=78)** | **p-value** |
| --- | --- | --- | --- | --- | --- |
| **Sex, n(%)**  **Female**  **Male** | 338 (54.7%)  318 (45.3%) | 194 (54.3%)  163 (45.7%) | 152 (56.9%)  115 (43.1%) | 38 (48.7%)  40 (51.3%) | 0.432 |
| **Age, mean±SD** | 47±14 | 48±13 | 46±14 | 48±15 | 0.218 |
| **NAS, n(%)**  **1-2 Points**  **3-4 Points**  **≥5 points** | 328 (46.7%)  202 (28.7%)  173 (24.6%) | 192 (53.6%)  101 (28.2%)  65 (18.2%) | 112 (41.9%)  80 (30.0%)  75 (28.1%) | 24 (30.8%)  21 (26.9%)  33 (42.3%) | <0.001 |
| **NAS, n(%)**  **1-4 Points**  **≥5 points** | 530 (75.4%)  173 (24.6%) | 293 (81.8%)  65 (18.2%) | 192 (71.9%)  75 (28.1%) | 45 (57.7%)  33 (42.3%) | <0.001 |
| **NAS, median (IQR)** | 3 (1-4) | 2 (1-4) | 3 (1-5) | 4 (2-5) | <0.001 |
| **Steatosis, %**  **Median (IQR)** | 40 (15-70) | 30 (10-60) | 40 (20-70) | 50 (29-70) | <0.001 |
| **NAS Steatosis, n(%)**  **Grade 1**  **Grade 2-3** | 344 (48.9%)  359 (51.1%) | 206 (57.5%)  152 (42.5%) | 114 (42.7%)  153 (57.3%) | 24 (30.8%)  54 (69.2%) | <0.001 |
| **NAS Steatosis, n(%)**  **Grade 1-2**  **Grade 3** | 521 (74.1%)  182 (25.9%) | 294 (82.1%)  64 (17.9%) | 175 (65.5%)  92 (34.5%) | 52 (66.7%)  26 (33.3%) | <0.001 |
| **NAS Inflammation, n(%)**  **Grade 0-1**  **Grade 2-3** | 639 (90.9%)  64 (9.1%) | 327 (91.3%)  31 (8.7%) | 244 (91.4%)  33 (8.6%) | 68 (87.2%)  10 (12.8%) | 0.481 |
| **NAS Ballooning, n(%)**  **Grade 0-1**  **Grade 2** | 606 (86.2%)  97 (13.8%) | 321 (89.7%)  37 (10.3%) | 226 (84.6%)  41 (15.4%) | 59 (75.6%)  19 (24.4%) | 0.003 |
| **Fibrosis, n(%)**  **Grade 0-2**  **Grade 3/4** | 622 (88.5%)  81 (11.5%) | 327 (91.3%)  31 (8.7%) | 236 (88.4%)  31 (11.6%) | 59 (75.6%)  19 (24.4%) | <0.001 |
| **Cirrhosis, n(%)**  **No**  **Yes** | 674 (95.9%)  29 (4.1%) | 347 (96.9%)  11 (3.1%) | 257 (96.3%)  10 (3.7%) | 70 (89.7%)  8 (10.3%) | 0.014 |
| **Diabetes, n(%)**  **No**  **Yes** | 479 (68.1%)  224 (31.9%) | 241 (67.3%)  117 (32.7%) | 186 (69.7%)  81 (30.3%) | 52 (66.7%)  26 (33.3%) | 0.789 |
| **BMI, median (IQR)** | 43.1 (30.6-50.4) | 43.5 (32.8-50.5) | 43.8 (31.2-51.0) | 32.0 (27.9-46.7) | 0.001 |
| **AST, median (IQR)** | 30 (23-44) | 28 (22-41) | 32 (24-47) | 37 (26-57) | <0.001 |
| **ALT, median (IQR)** | 40 (26-63) | 36 (24-55) | 42 (27-68) | 52 (34-82) | <0.001 |
| **GGT, median (IQR)** | 44 (27-84) | 43 (26-69) | 43 (26-91) | 48 (32-105) | 0.187 |

Abbreviations: NAS – Non-Alcoholic Fatty Liver Disease (NAFLD) Activity Score, BMI – Body Mass Index, AST – Aspartate Aminotransferase, ALT – Alanine Aminotransferase, GGT – Gamma Glutamyl Transferase. Statistical Analysis within tables S1-S3: Student’s t-test was used for group comparisons of normally distributed data (mean±SD), while Mann-Whitney-U test was applied when data was not normally distributed (median[IQR]). Pearson's chi-squared test or Fisher’s exact test were performed to conduct group comparisons of categorical variables.

**Suppl. Table S2:** Patients stratified according to their TM6SF2 status. Since only n=8 patients were found with the T/T variant we have grouped all patients with at least one T-allele together to be able for perform adequate statistical analyses.

|  | **C/C (n=588)** | **C/T or T/T (n=115)** | **p-value** |
| --- | --- | --- | --- |
| **NAS, n(%)**  **1-2 Points**  **3-4 Points**  **>= 5 points** | 286 (48.6%)  166 (28.2%)  136 (23.1%) | 42 (36.5%)  36 (31.3%)  37 (32.2%) | 0.038 |
| **NAS, n(%)**  **1-4 Points**  **≥5 points** | 452 (76.9%)  136 (23.1%) | 78 (67.8%)  37 (32.2%) | 0.039 |
| **NAS, median (IQR)** | 3 (1-4) | 4 (2-5) | 0.005 |
| **Steatosis, %, Median (IQR)** | 35 (10-70) | 50 (30-70) | <0.001 |
| **NAS Steatosis, n(%)**  **Grade 1**  **Grade 2-3** | 302 (51.4%)  286 (48.6%) | 42 (36.5%)  73 (63.5%) | 0.004 |
| **NAS Steatosis, n(%)**  **Grade 1-2**  **Grade 3** | 443 (75.3%)  145 (24.7%) | 78 (67.8%)  37 (32.2%) | 0.092 |
| **NAS Inflammation, n(%)**  **Grade 0-1**  **Grade 2-3** | 539 (91.7%)  49 (8.3%) | 100 (87.0%)  15 (13.0%) | 0.108 |
| **NAS Ballooning, n(%)**  **Grade 0-1**  **Grade 2** | 512 (87.1%)  76 (12.9%) | 94 (81.7%)  21 (18.3%) | 0.129 |
| **Fibrosis, n(%)**  **Grade 0-2**  **Grade 3/4** | 527 (89.6%)  61 (10.4%) | 95 (82.6%)  20 (17.4%) | 0.031 |
| **Cirrhosis, n(%)**  **No**  **Yes** | 566 (96.3%)  22 (3.7%) | 108 (93.9%)  7 (6.1%) | 0.247 |
| **Diabetes, n(%)**  **No**  **Yes** | 398 (67.7%)  190 (32.3%) | 81 (70.4%)  34 (29.6%) | 0.563 |
| **BMI, median (IQR)** | 43.6 (31.2-50.6) | 39.6 (28.7-48.9) | 0.020 |
| **AST, median (IQR)** | 30 (23-43) | 34 (25-48) | 0.040 |
| **ALT, median (IQR)** | 39 (25-60) | 43 (28-71) | 0.034 |
| **GGT, median (IQR)** | 43 (26-84) | 45 (28-85) | 0.585 |

Abbreviations: NAS – Non-Alcoholic Fatty Liver Disease (NAFLD) Activity Score, BMI – Body Mass Index, AST – Aspartate Aminotransferase, ALT – Alanine Aminotransferase, GGT – Gamma Glutamyl Transferase.

**Suppl. Table S3:** Patients stratified according to their HSD17B13 status.

|  | **T/T (n=398)** | **T/TA (n=250)** | **TA/TA (n=55)** | **p-value** |
| --- | --- | --- | --- | --- |
| **NAS, n(%)**  **1-2 Points**  **3-4 Points**  **≥ 5 points** | 192 (48.2%)  93 (23.4%)  113 (28.4%) | 117 (46.8%)  81 (32.4%)  52 (20.8%) | 19 (34.5%)  28 (50.9%)  8 (14.5%) | <0.001 |
| **NAS, n(%)**  **1-4 Points**  **≥5 points** | 285 (71.6%)  113 (28.4%) | 198 (79.2%)  52 (20.8%) | 47 (85.5%)  8 (14.5%) | 0.018 |
| **NAS, median (IQR)** | 3 (1-5) | 3 (1-4) | 3 (2-4) | 0.842 |
| **Steatosis, %**  **Median (IQR)** | 38 (15-70) | 40 (11-70) | 45 (20-70) | 0.296 |
| **NAS Steatosis, n(%)**  **Grade 1**  **Grade 2-3** | 200 (50.3%)  198 (49.7%) | 122 (48.8%)  128 (51.2%) | 22 (40.0%)  33 (60.0%) | 0.362 |
| **NAS Steatosis, n(%)**  **Grade 1-2**  **Grade 3** | 301 (75.6%)  97 (24.4%) | 184 (73.6%)  66 (26.4%) | 36 (65.5%)  19 (34.5%) | 0.265 |
| **NAS Inflammation,n(%)**  **Grade 0-1**  **Grade 2-3** | 356 (89.4%)  42 (10.6%) | 231 (92.4%)  19 (7.6%) | 52 (94.5%)  3 (5.5%) | 0.276 |
| **NAS Ballooning, n(%)**  **Grade 0-1**  **Grade 2** | 334 (83.9%)  64 (16.1%) | 222 (88.8%)  28 (11.2%) | 50 (90.9%)  5 (9.1%) | 0.123 |
| **Fibrosis, n(%)**  **Grade 0-2**  **Grade 3/4** | 348 (87.4%)  50 (12.6%) | 224 (89.6%)  26 (10.4%) | 50 (90.9%)  5 (9.1%) | 0.591 |
| **Cirrhosis, n(%)**  **No**  **Yes** | 379 (95.2%)  19 (4.8%) | 243 (97.2%)  7 (2.8%) | 52 (94.5%)  3 (5.5%) | 0.411 |
| **Diabetes, n(%)**  **No**  **Yes** | 268 (67.3%)  130 (32.7%) | 177 (70.8%)  73 (29.2%) | 34 (61.8%)  21 (38.2%) | 0.378 |
| **BMI, median (IQR)** | 41.8 (30.1-49.1) | 44.3 (31.2-50.6) | 43.8 (31.3-53.0) | 0.082 |
| **AST, median (IQR)** | 32 (23-45) | 29 (23-41) | 32 (24-48) | 0.089 |
| **ALT, median (IQR)** | 41 (26-65) | 38 (25-59) | 42 (27-61) | 0.313 |
| **GGT, median (IQR)** | 44 (26-88) | 43 (27-74) | 39 (26-84) | 0.778 |

Abbreviations: NAS – Non-Alcoholic Fatty Liver Disease (NAFLD) Activity Score, BMI – Body Mass Index, AST – Aspartate Aminotransferase, ALT – Alanine Aminotransferase, GGT – Gamma Glutamyl Transferase.

**Suppl. Figure S4:** AUC for predicting significant fibrosis (≥F2) using (A) baseline model (BL; age, sex, BMI, and diabetes), (B) BL + PNPLA3, (C) BL + HSD17B13 (D) BL + TM6SF2;

P-values: (B), (C), and (D) vs. BL model


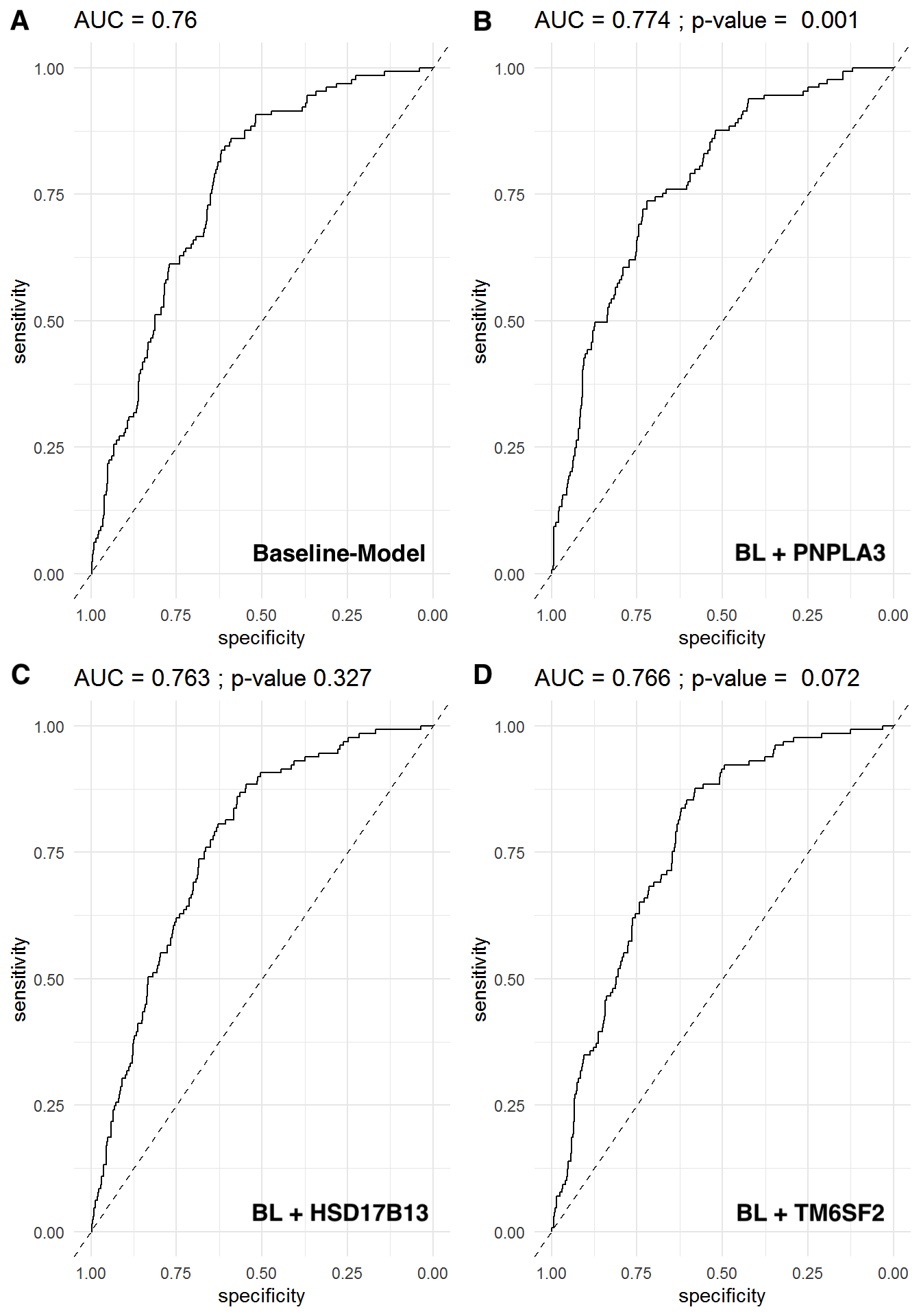


**Suppl. Figure S5:** AUC for predicting advanced fibrosis (≥F3) using (A) FIB-4 (B) FIB-4 + PNPLA3, (C) FIB-4 + HSD17B13 and (D) FIB-4 + TM6SF2;

P-values: (B), (C), and (D) vs. FIB-4 model).


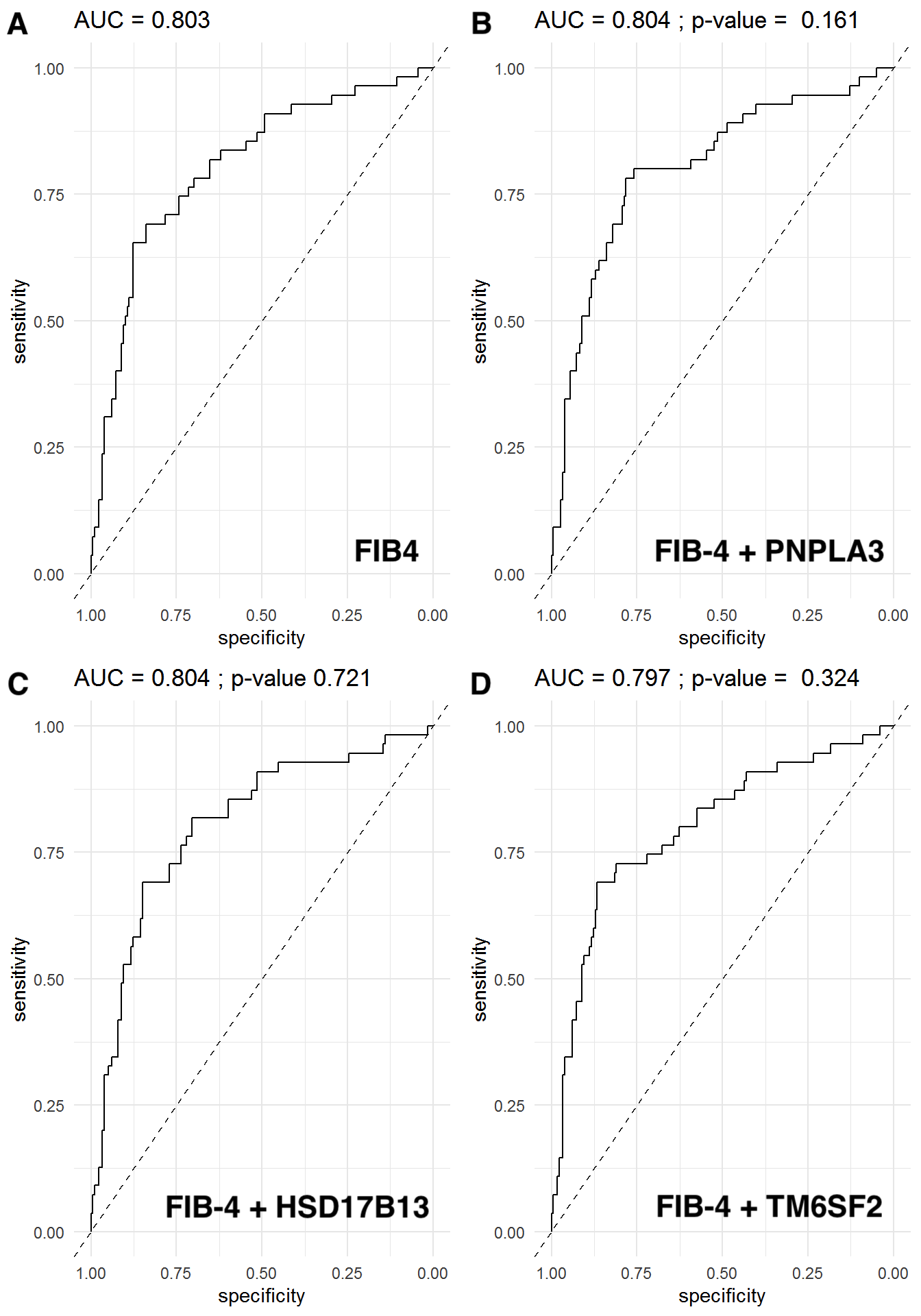


**Suppl. Figure S6:** AUC for predicting advanced fibrosis (≥F3) using (A) NAFLD-Fibrosis Score [NFS](B) NFS + PNPLA3, (C) NFS + HSD17B13 and (D) NFS + TM6SF2;

P-values: (B), (C), and (D) vs. NFS model).


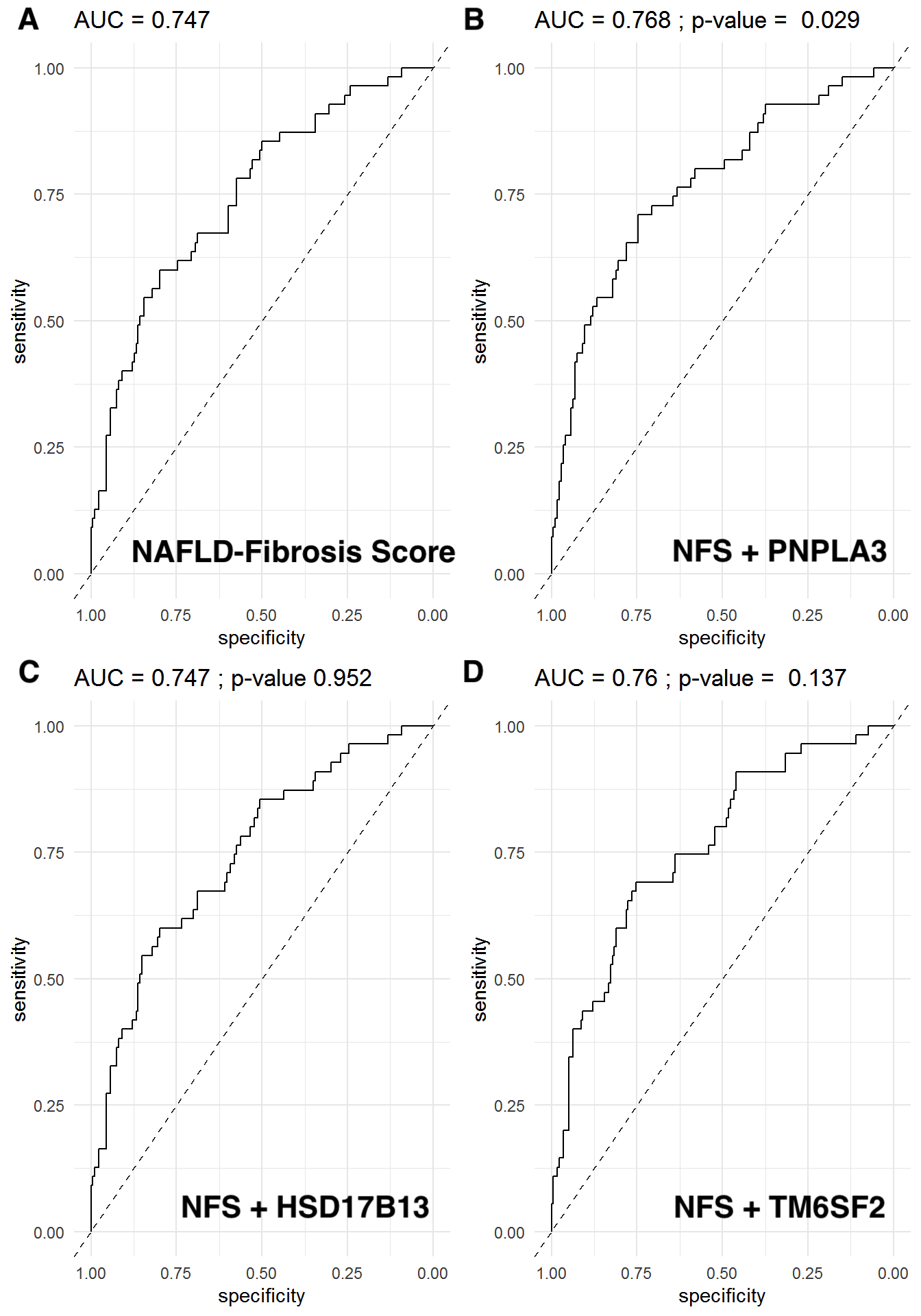

Supplement: Supplementary file 1 — Supplementary file1 (DOCX 438 kb) [file 12072_2021_10200_MOESM1_ESM.docx]
